# A Report of Two Difficult Ovariotomies

**Published:** 1890-05

**Authors:** J. J. Buchanan

**Affiliations:** Pittsburgh


### A REPORT OF TWO DIFFICULT OVARIOTOMIES.*

By J. J. BUCHANAN, M. D., PITTSBURGH.

The three specimens of ovarian cyst which I present for examination represent two ovariotomies which were interesting by reason of the difficulties of their execution, and in one instance the unusual position of the tumor in relation to a loop of the small intestine. All were intra-ligamentous, and all were successfully enucleated by the method of the late Dr. Miner, of Buffalo.

CASE I.—Operation December 17, 1889, at Mercy Hospital. This patient, a married woman, thirty-three years of age, without children, had noticed an abnormal enlargement of her abdomen for eight years. Of slow growth at first, this tumor had rapidly increased during the past year, and had been the cause of great pain. About one year before operation she had begun to resort to the use of morphine to relieve her pain, and had gradually increased the amount to eight grains per day, always taking a single dose. She had emaciated greatly, and had developed markedly the classical *facies ovariana* now so rarely ob-

*Read before the Allegheny County Medical Society, March 18, 1890.

served, thanks to early diagnosis and operation. The cyst at the time of operation was considerably larger than the pregnant uterus at term.

On the left side, above Poupart's ligament, could be felt a globular mass which proved to be the fundus uteri.

When the abdomen was opened the free surface of the cyst presented, and before evacuation by the trocar, everything appearing favorable.

When the contents had been partially evacuated, it was found that the cyst was implanted in the broad ligament, and its base extended over the entire width of the pelvis. After some omental adhesions had been tied off, a careful examination of the situation of the cyst was made. It had originated in the left ovary (as the position of the vessels subsequently showed) and had separated the folds of the left broad ligament, pushed its way behind the uterus, to which it was intimately attached, and imbedded itself deeply in the right broad ligament where its greatest development had taken place. A beginning of the enucleation was made by separating the peritoneal and fibrous investment from the body of the cyst at the fundus of the uterus. The circumcision of these external layers was then continued at about the same level, and the enucleation proceeded with as rapidly as the density of the tissues would permit. A pedicle was finally made at the left cornu of the uterus, which was tied, burned and dropped.

The tattered remains of the broad ligaments were brought together with a continuous silk suture, an aperture being left for the insertion of a glass drain into the cavity left by the growth. Several gallons of hot distilled water were used to flush the peritoneum and the wound cavity. The margins of the sac were stitched into the lower angle of the external wound and the incision closed.

The following day the patient developed an acute bronchitis, and her temperature on the second evening went up to 103¾°, with a pulse of 140. She was very ill for six days, and it was only by the persistent use of enemata of brandy and peptonized foods and large doses of carbonate of ammonia by the mouth that her strength was sustained. Her abdomen remained flat and the incision healed in the usual aseptic manner, the drainage tube being withdrawn on the third day.

On account of the aggravation of her cough by recumbency, she was encouraged to leave the bed on the eighth day, and on the twelfth day was walking about the room. Her recovery thereafter was uninterrupted. She has since menstruated for the first time for ten months, and since the first week after operation has taken no morphine.

CASE II.—Operation February 6th, 1890, at Mercy Hospital. This patient was also a married woman, 36 years of age. Three years ago she noticed a lump in her left iliac region, which gradually increased in size till it was much larger than a pregnant womb at term, having doubled in size in six months. The growth was painless, and the patient, at time of operation, in robust health.

Abdominal incision revealed the tumor completely covered in front and below by adherent omentum and a strip of small intestine, which was attached to the tumor vertically from the umbilicus to the pubes, and which disappeared behind the pubic bone. By enlarging the incision above the navel and to the pubes, room was made for manipulation. A large mass of omentum was lifted from the tumor and cut between ligatures. When this had been stripped from the tumor, a more satisfactory examination could be made. On either side of the vertical strip of small intestine, extended a thin vascular membrane, which on the left lost itself in the peritoneal investment of the tumor, and on the right was everywhere closely adherent to it. There is no doubt that this was the attenuated remains of the mesentery. The cyst was tapped high up on the left side, and a thick, yellow, ovarian fluid evacuated. As the cyst collapsed, its walls were found to pass to the lateral margins of the pelvic brim, and to be closely attached to the posterior surface of the uterus. Enucleation offered the only chance of extirpating the growth, and it was determined to make an effort to accomplish this. It was also a very serious question as to the best manner of dealing with the vertical strip of bowel which formed a sort of equator for the cyst. Its mesenteric attachment being obliterated, or rather spread out and amalgamated with the covering of the cyst, it was deemed advisable to begin the enucleation by a vertical incision through the serous coat of the cyst immediately to the left of and parallel with the strip of bowel; for on this side the covering seemed thinner. By lifting together the strip of bowel and the covering of the tumor to the right of it in a continuous layer, it was hoped that this pseudo-mesentery would afford sufficient blood-supply to preserve the vitality of the gut. The enucleation proved very tedious on account of the extent of surface involved, and the tenuity of the covering which was to form a mesentery for the gut and which it was therefore desired to preserve intact. This, however, proved impossible, and a large rent was made in the false mesentery.

When the enucleation was complete and the pedicle secured, an examination showed that the rent above mentioned had left about ten inches of intestine without mesenteric attachment. It then became a question whether a continuous suture of this rent or a resection of the bowel would be the better plan. The former was decided upon, and a continuous silk suture was applied to the whole length of the rent, in the hope that the middle of the strip of intestine would get sufficient blood-supply from anastomoses through the covering of the gut itself, and by new vessels thrown across the line of suture. Fortunately it so transpired.

The other ovary was then sought for and found to be the size of a large orange, and also intra-ligamentous. It was emptied, circumcised and enucleated; it had no pedicle. Its contents were heavily charged with oil globules.

The operation had now occupied the major part of two hours and it seemed hopeless to attempt any repair of the tattered remnants of the broad ligaments, even if the patient had been in condition to endure a continuance of the operation, which she was not, being in a condition of profound shock. A glass drain was therefore inserted, after profuse flushing of the cavity, and the wound closed and dressed with double cyanide gauze. For six or eight hours after being put to bed, she lay almost pulseless, and required repeated hypodermic injections of whiskey and enemata of hot salt water to revive her. After reaction was established, her recovery was uninterrupted; her bowels were moved on the sixth day by repeated doses of Rochelle salts. A rise of temperature above the normal was noted on but one occasion, prior to the movement of her bowels on the sixth day, when the thermometer showed 100.5°. She walked out of her room on the twelfth day, and on the sixteenth left the hospital.

I have been unable to satisfy myself as to the manner in which this tumor and the small intestine assumed the relations which existed between them. Two explanations suggest themselves: the first that the subserous tumor pushed its way behind the prevertebral peritoneum and insinuated itself between the layers of the mesentery; the second, that the intra-ligamentous tumor, when small, contracted a broad adhesion to the bowel and mesentery, which latter, as the tumor grew, became greatly attenuated, as it was widely stretched and firmly glued to the surface of the growth. A more deliberate and careful examination after the enucleation of the large cyst might have thrown light upon this question, but the condition of the patient rendered it hazardous.

An interesting point in this case is that, had this woman been tapped at the usual site, the trocar would certainly have perforated the bowel.

In closing this report I cannot refrain from calling attention to the fact that these operations were both done in a general hospital, and expressing my conviction, as I did in this Society five years ago, that no reason in the world exists for fencing off the abdomen from the domain of the general surgeon; and further, that special abdominal hospitals exist for the convenience and profit of their owners, and are by no means necessary for the safety of the patients.

